# Promoting Innovative Performance in Multidisciplinary Teams: The Roles of Paradoxical Leadership and Team Perspective Taking

**DOI:** 10.3389/fpsyg.2018.01083

**Published:** 2018-07-02

**Authors:** Quan Li, Zhuolin She, Baiyin Yang

**Affiliations:** School of Economics and Management, Tsinghua University, Beijing, China

**Keywords:** expertise diversity, paradoxical leadership, team perspective taking, team innovative performance, team innovation paradox

## Abstract

Although many researches recognize the role of team expertise diversity in providing different ideas, it remains unclear how and under which conditions these various ideas are elaborated and integrated to fuel team innovation. To address this question, we develop a model theorizing that paradoxical leadership helps diverse teams overcome the differentiating-integrating paradox to promote innovation. Moreover, we further theorize that paradoxical leadership will cultivate perspective taking among team members. Analyses of the multi-time and multi-source data from 98 teams suggest that teams with expertise diversity exhibit better innovative performance when paradoxical leadership is prevalent. Furthermore, team perspective taking mediates the positive moderating effects of paradoxical leadership on the relationship between expertise diversity and innovative performance. Through these analyses, this study not only addresses the innovation paradox of expertise diverse teams from the perspective of leader influence, but also enriches the understanding of the effects of paradoxical leadership.

## Introduction

As the business environment becomes more challenging, firms increasingly employ work teams composed of members with diverse functional or educational backgrounds to fuel innovation. Nevertheless, several empirical findings suggested expertise diversified teams do not easily promote innovative performance (Bassett-Jones, 2005; Horwitz and Horwitz, 2007; Guillaume et al., 2017). The major reason is that such teams inevitably encounter a differentiation-integration paradox (van Knippenberg, 2017). Specifically, expertise diversity will increase the opportunity to generate various original ideas to meet the requirement for differentiation (Andriopoulos and Lewis, 2009), while also raising the likelihood that group members will be dissatisfied and fail to embrace others’ opinions (Milliken and Martins, 1996). This inhibits the effective integration of the information and knowledge obtained from others (van Knippenberg et al., 2004).

Accordingly, many previous studies focus on identifying the conditions under which diverse teams can satisfy the differentiation-integration demands of innovation (Joshi and Roh, 2009; Guillaume et al., 2017), such as team-oriented HR practices (Chi et al., 2009), minority dissent (De Dreu and West, 2001), team’s climate for innovation (Somech and Drach-Zahavy, 2013), and team open-mindedness norms (Mitchell and Boyle, 2015). However, the knowledge of when and how expertise diversity promotes team innovation is still fragmentary and leaves many questions unanswered (Guillaume et al., 2017; van Knippenberg, 2017). For example, there is little knowledge on how leadership affects the relationship between expertise diversity and team innovation (van Knippenberg, 2017). Hence, this study aims to fill this gap by analyzing whether and how (i.e., the processes through which) paradoxical leaders assist expertise diverse teams to achieve their full potential in pursuit of innovation.

Paradoxical leadership is defined as “seemingly competing, yet interrelated, behaviors to meet structural and follower demands simultaneously and over time” (Zhang et al., 2015, p. 539). The central idea of paradoxical leadership is that the leader adopts “both-and” approaches that behaviorally accept and integrate competing demands simultaneously over time to harness the intention within the paradox (Zhang et al., 2015; Waldman and Bowen, 2016). Accordingly, we propose that paradoxical leaders may respect every team member’s viewpoint, encourage all members to voice differentiated ideas and opinions. Meanwhile, they may also promote team information integration and offer necessary instructions and guidance to achieve innovative goals. Combining these managerial practices, paradoxical leaders could lead diverse team members to meet the differentiation and integration requirements of innovation. To further understand how paradoxical leaders facilitate innovation within expertise diverse teams, it is important to consider the role of team processes (Ilgen et al., 2005). We thus incorporate team perspective taking into the model and suggest it as an intervening factor to explain the moderating effects of paradoxical leadership. Team perspective taking is an emergent team-level cognitive process that entails sharing, communicating, and integrating each team member’s views (Parker et al., 2008). We posit that, under the influence of paradoxical leaders, team members may learn to share and integrate various perspectives, which can facilitate the elaboration and integration process, thus enhancing team innovation.

**FIGURE 1:**
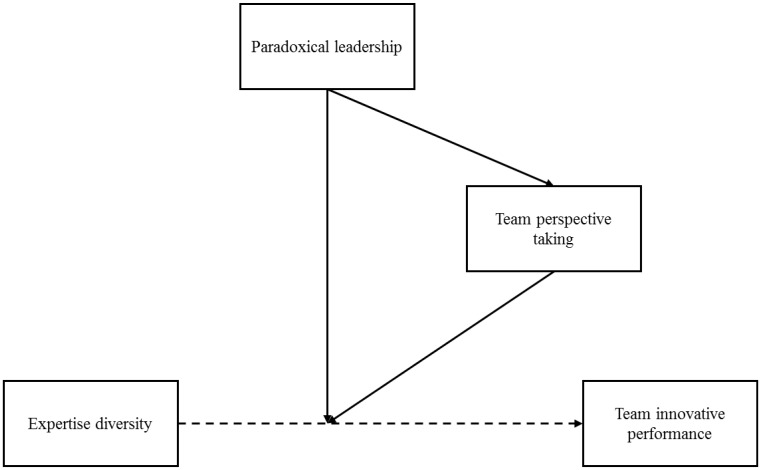
The proposed model.

In sum, this study focuses on paradoxical leadership as a driving factor to deal with the differentiation-integration paradox inherent in expertise diverse teams. **Figure 1** presents our proposed research model. Through a series of investigations, we aim to offer three specific contributions to the literature. First, we respond to the research call of exploring approaches to reconcile the team innovation paradox and provide new insight into how paradoxical leaders help expertise diverse teams meet the differentiation-integration requirements. Second, with respect to the paradoxical leadership literature, we are among the first to offer an in-depth understanding of the role of paradoxical leadership in the team context, specifically for expertise diverse teams, which extends this stream of research from the individual level to the team level. Third, we also demonstrate the moderating role of team perspective taking in fueling diverse teams’ innovation, and further elaborate on it as an important mechanism through which paradoxical leaders exert influence. Thus, this study also enriches our understanding of the role of team perspective taking.

## Theoretical Background and Hypotheses

### The Innovation Paradox in Expertise Diversified Teams

The innovation process contains two “related and identical” stages, namely idea generation and idea implementation (West and Farr, 1989; Amabile, 1996; Anderson et al., 2014). Idea generation requires more divergent facilitators that enable teams to think outside the box and constantly search for new possibilities, while idea implementation requires more convergent drivers that help to promote novel and useful ideas through well-established channels and integrate the innovation plan into an organizational setting (West, 2002). However, the conditions facilitating new idea creation may impede the idea-implementation process, and vice versa (Frost and Egri, 1991; Hargadon and Douglas, 2001; Gong et al., 2013), suggesting an “innovation paradox,” reflected by the potential tension between the two innovation processes (Miron-Spektor et al., 2011; Carnabuci and Diószegi, 2015). Therefore, teams must balance the conflicting differentiation-integration demands in the innovation process and engage in both creation and implementation activities.

However, this is not easy for expertise diversified teams. van der Vegt and Bunderson (2005) define expertise diversity as differences in the knowledge and skill domains within a group due to their education and work experiences. On the one hand, members of expertise diverse teams are more likely to contribute unique opinions and perspectives and give suggestions and comments from different angles, thus facilitating idea generation (Shin et al., 2012). On the other hand, team members are also likely to categorize themselves based on categorical identities. In this case, they may perceive ideas from members with different functional backgrounds as less valuable and regard dissenting opinions as threats to their own identities (Jackson and Joshi, 2004; van Knippenberg et al., 2004), which then prevents the team from integrating diverse ideas and ultimately hinders idea implementation. Given that diverse teams are relatively difficult to self-regulate, researchers call for more attention to situational settings that may guide diverse teams out of the innovation paradox (Zhou and Hoever, 2014; Guillaume et al., 2017).

### The Moderating Role of Paradoxical Leadership

In the organizational context, leaders always encounter various management paradoxes (Lavine, 2014), such as the balance between control and authorization, efficiency and flexibility, individualism and collectivism. Conventional leadership contingency theory holds that faced with the management paradox, leaders are expected to make the best decision between the two; for example, choosing authorization or control (Waldman and Bowen, 2016). However, according to Smith and Lewis (2011), such a decision is favorable only in the short term. To achieve long-term performance, leaders should accept and reconcile paradoxes and coordinate conflicting needs well (Smith and Lewis, 2011). Based on two empirical studies, Zhang et al. (2015) propose the construct of paradoxical leadership and use “both-and” terminology to describe its five characteristics: (1) the combination of being self-centered and other-centered, that is paradoxical leaders maintain the central influence while simultaneously sharing concerns and respects for followers; (2) maintaining both distance and closeness, that is the leader keeps vertical structural relationships with followers when addressing work issues while simultaneously forming interpersonal bonds with them; (3) treating subordinates uniformly while allowing individualization, which means the leader assigns homogeneous positions for followers without displaying favoritism and simultaneously takes individual considerations into account; (4) enforcing work requirements while allowing flexibility, that is the leader sets requirements to regulate follower behaviors in work processes and simultaneously gives followers discretion to act flexibly; (5) maintaining decision control while allowing autonomy, which means the leader uses authority in decision making to ensure work outcomes and simultaneously gives followers appropriate autonomy.

Some researchers find that paradoxical leadership plays an important role in organizations. Based on a case study of five enterprises, Lewis and Smith (2014) find that paradoxical leadership brings both flexibility and stability, thus helping firms better adapt to dynamic external conditions. Empirical studies by Zhang et al. (2015) suggest that paradoxical leaders endow subordinates with proficiency, adaptivity, and proactivity, which benefits their short- and long-term development. In this regard, we argue below that paradoxical leadership is particularly promising as a way to address the differentiating-integrating paradox of innovation inherent in expertise diverse teams.

According to motivated information processing theory (De Dreu et al., 2008; Nijstad and De Dreu, 2012), to increase the magnitude and depth of innovative information processing, teams should function in contexts that not only include distinctly different perspectives and viewpoints, but also ensure that information can be shared and integrated as a collective orientation. Paradoxical leaders respect every team member’s views and simultaneously encourage them to respond to other members’ differing ideas and opinions (Zhang et al., 2015). Under the paradoxical leaders’ influence, team members may learn to be open to multiple options and perspectives, consider the range of alternate perspectives from other members, and be willing to apply them to collective tasks. Therefore, although expertise diversity is likely to yield various perspectives on work tasks, these differences may yield synergistic effects and thereby improve team innovation given the catalytic effects of paradoxical leaders.

Furthermore, paradoxical leaders provide followers with the discretion to utilize their personal strengths and capabilities while emphasizing team norms and standards (Zhang et al., 2015). That is, teams with expertise diversity can explore new possibilities under an established structure (i.e., clear performance standards and goals) set by the paradoxical leader, which can ensure concordance between creative outputs and organizational demands. As such, when executing innovation plans, they are less likely to deviate from shared goals, leading to more efficient promotion and implementation of innovative ideas. Therefore, we propose the following:

Hypothesis 1. Paradoxical leadership moderates the relationship between expertise diversity and team innovative performance such that this relationship is more positive when paradoxical leadership is prevalent.

### The Role of Team Perspective Taking

To shed light on the possible mechanisms underlying the above hypothesis, we further propose that team perspective taking mediates the moderating effects of paradoxical leadership on the relationship between expertise diversity and team innovative performance. In the following, we first explain why paradoxical leadership facilitates team perspective taking and then interpret why team perspective taking moderates the impact of expertise diversity on team innovative performance.

Team perspective taking is a collective cognitive process, through which team members strive to understand the world from other members’ viewpoints (Hoever et al., 2012; Li, 2016). As information processing theory (Griffin, 1983) pointed out, the team’s cognitive and behavioral process is a result of processing and interpreting social cues, where leaders serve as primary sources of social information to guide team members’ thoughts and behaviors (Lord and Maher, 2002). Therefore, we expect that the direct leader’s paradoxical behaviors will promote team perspective taking.

First, paradoxical leaders treat all team members with respect and appreciate the viewpoints and contributions of others (Zhang et al., 2015). By observing paradoxical leaders’ behaviors at work, team members may mimic these behaviors and try to comprehend and appreciate other teammates’ perspectives (Waldman and Bowen, 2016). Second, paradoxical leaders also cultivate a bounded discretionary work climate (Zhang et al., 2015), which encourages collaboration and positive interactions within the team, thereby creating favorable conditions for the development of team perspective taking. Parker and Axtell (2001) find that frequent interactions enable team members to understand others’ perspectives and the reasons for these perspectives. Therefore, we propose the following hypothesis:

Hypothesis 2a. Paradoxical leadership is positively associated with team perspective taking.

Motivated information processing theory suggests that groups perform cognitive tasks well (e.g., decision making, problem solving, or innovation) when team members hold non-redundant knowledge and information, and are simultaneously willing to systematically process information with collective efforts. To translate multiple novel ideas into tangible entities, team members need to integrate different perspectives without prejudice and make joint efforts to put forward innovative plans (van der Vegt and Bunderson, 2005). Therefore, we propose that team perspective taking moderates the effects of expertise diversity on team innovation performance.

Teams with high perspective taking are more likely to process and integrate diverse viewpoints to fuel innovation. Specifically, team perspective taking can encourage team members to share and discuss diverse options without prejudice and help them improve and refine creative ideas collectively (Hoever et al., 2012). Consequently, these diversified viewpoints will be elaborated as more feasible and reliable solutions, and team members may be willing to devote more time and energy to idea implementation. In support of our argument, previous studies find that team members who adopt the viewpoints of their coworkers are more likely to translate their creative ideas into tangible products (Purser et al., 1992).

Conversely, when team diversity is salient and team perspective taking is weak, members devote more attention to their own ideas rather than seeking feedback or comments from other members, thus causing a lack of collectivity. Empirical evidence shows that multidisciplinary teams with low levels of team perspective taking could show resistance to accepting information and perspectives from other members (Hoever et al., 2012). Failing to see the value of others’ ideas can increase the risk of making mistakes and decrease the efficiency of idea promotion and implementation, which then negatively relates to team innovation (Liedtka, 2015). Therefore, we propose:

Hypothesis 2b. Team perspective taking moderates the effects of expertise diversity on team innovative performance such that expertise diversity has a more positive effect on innovative performance for teams with a high level of team perspective taking.

The preceding two hypotheses propose that paradoxical leadership stimulates team perspective taking (Hypothesis 2a) and that team perspective taking moderates the association between expertise diversity and team innovative performance (Hypothesis 2b). Taken together, these two hypotheses predict that team perspective taking mediates the moderating effect of paradoxical leadership on the association between expertise diversity and team innovative performance, which is a case of mediated moderation (Edwards and Lambert, 2007). In sum, we predict the following:

Hypothesis 2c. Team perspective taking mediates the moderating effect of paradoxical leadership on the association between expertise diversity and team innovative performance.

## Materials and Methods

### Participants and Procedure

We collected data from two financial research centers of a state-owned bank located in Beijing and Shanghai, China. The main functions of the teams within these centers are to research and design new financial products and services. The company creates teams by assembling individuals with a range of relevant expertise, such as analysts, data-bank specialists, and market specialists. We invited team leaders and their subordinates to participate in our survey and guaranteed that their participation was totally voluntary and that their private information was confidential. Only teams with both supervisors and subordinates agreeing to take part were instructed on how to complete the survey.

Since common source data may inflate the correlations between variables and result in misleading conclusions, we collected data from different sources and at two different times. In the first wave, subordinates were invited to fill out questionnaires containing two moderator variables (i.e., paradoxical leadership and team perspective taking) and demographic questions, because subordinates have more opportunities to observe their leaders’ behaviors (Kim and Yukl, 1995) and can evaluate team cognitive process more accurately (Chan, 1998). At time 1, we collected a sample of 114 teams (523 subordinates) with response rates of 76 and 83% at the team and individual levels, respectively.

As Podsakoff et al. (2003) suggest, it is essential to measure dependent variables in the final wave to improve correlation validity considering the potential causal relationships among the variables. Hence, in the second wave (1 month later), we distributed questionnaires with the dependent variable (team innovative performance), to the supervisors because they have more comprehensive and objective judgments of team performance (Borman, 2017). After dropping 16 teams (63 subordinates) whose supervisors had not completed the questionnaires, our final dataset included 98 teams (460 subordinates) with overall response rates of 65 and 73% at the team and individual levels, respectively. The teams ranged from 3 to 9 members (*mean* = 5; *SD* = 1.18). The average age of team members was 33.27, and 53% of them were male. Moreover, 71% of team members had a bachelor’s degree and the others a master’s degree or higher.

### Measures

All variables we measured were from validated scales and all items were assessed on a six-point Likert-type scale ranging from 1 (strongly disagree) to 6 (strongly agree).

#### Expertise Diversity

Following the literature (van Knippenberg and Schippers, 2007; Li, 2016), we measured expertise diversity by calculating the educational disciplinary area and functional differentiation of team members. We divided educational specializations into five categories: arts, sciences, engineering, business and economics, and law. Functional backgrounds included industry analysis, investment product design, customer service, information technology, and operational management. We computed functional and educational diversity at the specialization level using Blau’s (1977) formula, 1 - Σ*pi*^2^, where *pi* is the proportion of a group in the *i*th category. We then took the average educational and functional diversity scores to capture the overall level of expertise diversity, where a higher index score indicates higher levels of expertise diversity among team members.

#### Team Perspective Taking

Team members rated this construct on a four-item scale developed by Grant and Berry (2011), which we then aggregated at the team level via the referent-shift model (Chan, 1998) (e.g., on the job, we frequently try to take other team members’ perspectives). Agreement among team members’ ratings had a mean RWG of 0.82, an ICC (1) of 0.18 (*p* < 0.001), and an ICC (2) of 0.52, suggesting it was appropriate to aggregate measures of perspective taking at the team level.

#### Paradoxical Leadership

Team members rated the 22-item scale developed by Zhang et al. (2015) (e.g., “Uses a fair approach to treat all subordinates uniformly, but also treats them as individuals”). Agreement among team members’ ratings had a mean RWG of.85, an ICC (1) of.12 (*p* < 0.001), and an ICC (2) of 0.40, supporting the aggregation of responses at the team level.

#### Team Innovative Performance

Because obtaining an objective innovation outcome measure is relatively difficult, Barrick et al. (1998) suggest using subjective judgments as an alternative. Therefore, team leaders rated the four-item team innovative performance scale developed by Anderson and West (1998) (e.g., “Team members often produce new services, methods or procedures”).

#### Control Variables

We included team size, tenure diversity, age diversity, and gender diversity as controls because prior work suggests that these variables are related to interpersonal contacts, knowledge bases, and performance (Ancona and Caldwell, 1992). We used the coefficient of variation (standard deviation divided by the mean) for continuous demographic variables—team tenure and age—and Blau’s (1977) index for gender. Moreover, to control for any potential confounding effects of company-level factors, we created a dummy variable (1, Center in Beijing; 0, Center in Shanghai) and controlled for it in our regression analysis.

### Confirmatory Factor Analysis

Given the nested nature of our dataset, we conducted a multilevel confirmatory factor analysis to assess the factor structure of the three variables (i.e., team perspective, paradoxical leadership, and team innovative performance) on the within and between levels by using Mplus 7.0 software (Muthén and Muthen, 2012). The statistical indexes for the hypothesized three-factor model reveal an adequate level of model fit [χ^2^(700) = 1001.82, χ^2^/*df* = 1.43, CFI = 0.90, TLI = 0.89, RMSEA = 0.03, SRMR for within = 0.05, SRMR for between = 0.10]. As **Table 1** shows, the three-factor model yields a higher degree of model fit than the two- (Δχ^2^ = 78.19, Δ*df* = 3, *p* < 0.001) and single-factor models (Δχ^2^ = 345.22, Δ*df* = 4, *p* < 0.001). Taken together, these results suggest that the three variables are distinct.

**Table 1:** Descriptive statistics and simple correlations.

Variables	*Mean*	*SD*	1	2	3	4	5	6	7	8	9
(1) Center in Beijing	0.49	0.50									
(2) Team size	4.69	1.18	0.24^*^								
(3) Team gender diversity	0.32	0.19	0.44^**^	0.32^**^							
(4) Team age diversity	0.19	0.09	−0.23^*^	−0.04	0.01						
(5) Team tenure diversity	0.55	0.28	0.04	0.14	0.13	0.15					
(6) Team expertise diversity	0.58	0.16	0.41^**^	0.21^*^	0.23^∗^	−0.06	−0.05				
(7) Paradoxical leadership	4.89	0.63	−0.14	0.12	0.05	0.04	−0.14	0.18	(0.96)		
(8) Team perspective taking	4.62	0.82	−0.11	0.16	0.06	−0.12	−0.09	0.15	0.50^***^	(0.86)	
(9) Team innovative performance	4.50	0.84	0.04	0.19	0.11	−0.03	−0.04	0.17	0.30^**^	0.63^***^	(0.85)

*N = 98 work teams.*^∗^*p < 0.05,*^∗∗^*p < 0.01,*^∗∗∗^*p < 0.001; two-tailed test. The numbers in parentheses on the diagonal are Cronbach’s alpha estimates.*

### Analysis

To test our hypotheses, we conducted linear regression analyses. Following Aiken and West (1991), we mean centered all predicting variables prior to creating the product terms. Moreover, we analyzed all interactions through simple slope analysis and plotted these interaction effects for 1 SD above and below the mean of the moderators (i.e., paradoxical leadership and team perspective taking). In terms of mediated moderation effects (Hypothesis 2c), we used moderated path analysis following Edwards and Lambert (2007) and applied parametric bootstrapping to test the significance of the indirect effect (Selig and Preacher, 2008).

## Results

**Table 2** presents the descriptive statistics, internal consistency reliabilities, and inter-correlations of all variables. The reliabilities of the measured variables exceed 0.80, providing strong evidence of internal consistency.

**Table 2:** Confirmatory factor analysis results.

Model	χ^2^	*df*	χ^2^/*df*	CFI	TLI	RMSEA	SRMR for within	SRMR for between
One-factor model (PL, PT, and TIP combined)	1347.04	704	1.91	0.79	0.77	0.05	0.05	0.10
Two-factor model (PL and PT combined)	1080.01	703	1.54	0.88	0.87	0.03	0.05	0.10
Three-factor model	1001.82	700	1.43	0.90	0.89	0.03	0.05	0.10

*PL, paradoxical leadership; PT, perspective taking; TIP, team innovative performance*.

**FIGURE 2:**
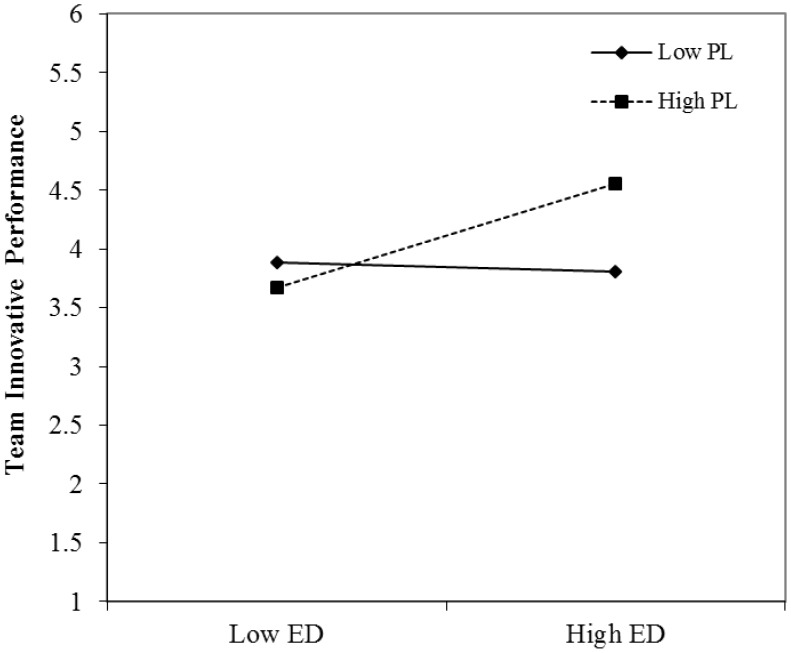
Interactive effect of expertise diversity and paradoxical leadership on team innovative performance. ED, expertise diversity; PL, paradoxical leadership.

Hypothesis 1 predicts a positive moderating effect of paradoxical leadership on the relationship between expertise diversity and team innovative performance. As Model 5 of **Table 3** shows, the regression coefficients for the interactions of paradoxical leadership and expertise diversity remain significant (β = 2.43, *p* < 0.01) after entering all control variables. **Figure 2** presents the interaction effects. The simple slope test results show that team expertise diversity is positively related to team innovative performance when leader paradoxical behaviors are prevalent (β = 2.40, *p* < 0.01), but the relationship between team expertise diversity and team innovative performance is not significant when these behaviors are less prevalent (β = −0.64, *p* > 0.05), thus supporting Hypothesis 1.

**Table 3:** Results of regression analysis.

Variables	Perspective taking		Team innovative performance				
	Model 1	Model 2	Model 3	Model 4	Model 5	Model 6	Model 7
**Control variables**							
Center in Beijing	−0.40^*^	−0.31	−0.06	0.18	−0.05	0.19	0.18
Team size	0.13	0.08	0.13	0.04	0.09	−0.00	0.00
Gender diversity	0.53	0.30	0.35	0.01	0.34	0.16	0.17
Age diversity	−1.44	−1.58	−0.20	0.76	−0.44	0.42	0.44
Tenure diversity	−0.30	−0.05	−0.21	−0.02	0.01	0.19	0.17
**Main effects**							
Expertise diversity (ED)		0.51		0.09	0.88	0.32	0.37
Paradoxical leadership (PL)		0.59^***^		−0.04	0.32^*^		−0.04
Perspective taking (PT)				0.66^***^		0.60^***^	0.61^***^
**Two-way interactions**							
ED × PL					2.43^**^		0.22
ED × PT						1.85^***^	1.76^**^
*R*^2^	0.10	0.30	0.05	0.42	0.20	0.50	0.51
Adjusted *R*^2^	0.05	0.25	0.00	0.37	0.13	0.46	0.45

*N = 98 work teams.*^∗^*p < 0.05,*^∗∗^*p < 0.01,*^∗∗∗^*p < 0.001; two-tailed test. All regression coefficients are unstandardized.*

Hypothesis 2a posits that paradoxical leadership positively influences team perspective taking. As Model 2 in **Table 3** shows, paradoxical leadership is positively associated with the team perspective (β = 0.59, *p* < 0.001) after controlling for the effects of confounding variables, thus supporting Hypothesis 2a.

**FIGURE 3:**
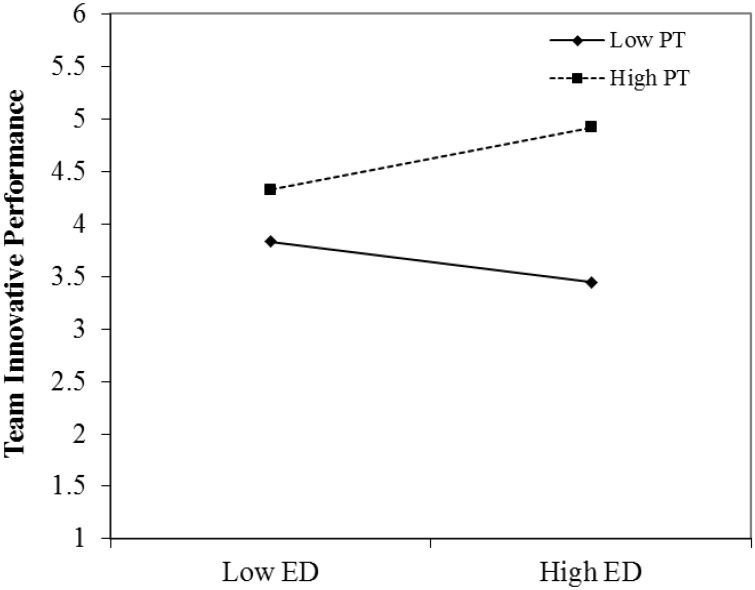
Interactive effect of expertise diversity and team perspective taking on team innovative performance. ED, expertise diversity; PT, perspective taking.

Hypothesis 2b proposes that team perspective taking moderates the association between expertise diversity and team innovative performance. As Model 6 in **Table 3** shows, the interaction term between team expertise diversity and team perspective taking positively related to team innovative performance (β = 1.85, *p* < 0.001). **Figure 3** depicts the interaction pattern. The results of a simple slope test show that the association between expertise diversity and team innovative performance is positive and significant (β = 1.84, *p* < 0.01) when teams exhibit high levels of perspective taking. Conversely, it is negative and significant in the presence of low levels of perspective taking (β = −1.20, *p* < 0.05), thus supporting Hypothesis 2b.

Hypothesis 2c proposes a mediated moderation model, wherein team perspective taking mediates the moderating effects of paradoxical leadership on the relationship between expertise diversity and team innovative performance. As Model 7 in **Table 3** indicates, when the model includes the interaction between team perspective taking and expertise diversity, the moderating effect of paradoxical leadership becomes insignificant (β = 0.22, *p* > 0.05), whereas that of team perspective taking remains significant (β = 1.76, *p* < 0.01), providing preliminary support for Hypothesis 2c. To further test whether including the moderating effects of team perspective taking significantly reduced the moderating effect of paradoxical leadership, we conducted a Monte Carlo simulation using an online parametric bootstrap procedure developed by Selig and Preacher (2008). The results show that the indirect effect size is 1.04 and the 95% confidence interval derived from the bootstrap analysis excludes zero [0.31, 1.93]. These findings support Hypothesis 2c.

## Discussion

Built on motivated information processing theory and the paradoxical leadership literature, we offer theoretical and empirical accounts of how paradoxical leadership assists expertise diverse teams in getting out of the innovation paradox. Using data from a sample of 98 teams, we demonstrate that expertise diverse teams achieved better innovative performance under the guidance of a more paradoxical supervisor. We also find that team perspective taking mediates the positive moderating effects of paradoxical leadership on the relationship between expertise diversity and team innovative performance. In doing so, this study opens up new avenues for future research.

### Theoretical Contributions

The current study makes three theoretical contributions. First, heeding van Knippenberg’s (2017) call to explore how leadership affects the relationship between expertise diversity and team outcomes, we provide a theoretical rationale and find empirical support showing that paradoxical leadership is particularly important for expertise diverse teams to fuel innovation. Although studies increasingly highlight the importance of leaders in promoting team innovation (Somech, 2006; Shin and Zhou, 2007), the literature provides scant evidence on how leaders assist a diversified team to solve the inherent innovation paradox. If the leader overemphasizes uniformity and compliance, it is difficult for team members to put forward diverse ideas, and even results in a conservative team mindset (De Hoogh et al., 2015). In contrast, teams with leaders who forego legitimate leader responsibilities, such as laissez-faire leaders who avoid confronting problems by ignoring followers’ needs (Yukl, 2010), may motivate team members to generate various opinions, but team members are less likely to finish their work in an orderly manner and cooperate with each other (Hinkin and Schriesheim, 2008; Wong and Giessner, 2016). In this study, we demonstrate that paradoxical leadership can effectively consider both the differentiation and integration challenges of innovation and help diverse teams meet the two seemingly contradictory demands through “both-and” management strategies. Hence, our study not only deepens our knowledge about the innovation paradox in diverse teams, but also enriches current research about the conditions under which teams may succeed in leveraging the innovation potential in expertise diversity.

Second, to the best of our knowledge, our study is the first in the paradoxical leadership literature that empirically investigates its effects at the team level. Thus, we add to a growing understanding of its effects beyond the individual level. While theoretical discussions on the concept of paradoxical leadership attract much attention (Lavine, 2014; Waldman and Bowen, 2016), this field contains limited empirical studies, which attempt only to investigate the effects of paradoxical leadership on subordinates. For example, Zhang et al. (2015) report that supervisors’ paradoxical behaviors positively predict subordinates’ proficiency, adaptivity, and proactivity. Tripathi (2017) shows that paradoxical leadership enhances subordinates’ work engagement. She and Li (2017) find that paradoxical leadership is positively related to subordinates’ job performance, mediated by subordinates’ leader identification. However, we know little about the effects of paradoxical leadership at the team level. Accordingly, based on an empirical study on teams, the current findings suggest that paradoxical leadership is a promising way to address the innovation paradox that expertise diversified teams face. By building this integrated model, our study expands the research into paradoxical leadership from the individual to the team level, which provides important insights into the effects of paradoxical leadership.

Third, our study further contributes to the current literature by documenting the importance of team cognitive processes in addressing the innovation paradox. In particular, team perspective taking helps teams embrace and evaluate various ideas comprehensively, thus facilitating the integration of diverse perspectives (Hoever et al., 2012). In doing so, we add to the previous literature on how team processes, such as innovation team climate (Somech and Drach-Zahavy, 2013) and team open-mindedness norms (Mitchell and Boyle, 2015), support innovation in diverse teams. Further, we also introduce team perspective taking as a key mechanism explaining the moderating effects of paradoxical leadership on diverse teams’ innovative performance. It extends Zhang’s et al. (2015) line of thinking and responds to her call to investigate the influencing mechanisms of paradoxical leadership in group processes and outcomes. However, other important mediators in this process may exist. It is possible that an inclusive team climate may also explain the moderating effects of paradoxical leadership because the “both-and” strategies of paradoxical leaders would help promote integration of differences (Zhang et al., 2015), the main characteristic of team inclusive climate. Future research could explore these questions.

### Practical Implications

The findings also have important implications for organizational management practice. First, our study indicates that paradoxical leadership can help diverse teams overcome the differentiating-integrating paradox to promote innovation. Since firms are increasingly turning to the use of diverse teams, the importance of paradoxical leadership as a means to unlock the innovation potential inherent in expertise diverse teams is also bound to increase. Therefore, it is important for organizations to cultivate paradoxical leader behaviors. On the one hand, organizations could identify qualified individuals who exhibit paradoxical leader behaviors and consider them as potential leaders of expertise diverse teams. Firms could do this by requiring candidates to finish relevant questionnaires or by assessing their paradoxical management abilities through leaderless group discussions during selection and recruitment. On the other hand, leaders of teams with high expertise diversity should be encouraged to think paradoxically and adopt “both-and” approaches to foster innovative performance. Specifically, organizations could provide various training opportunities focusing largely on paradoxical thinking and actions. Some training strategies such as learning from others’ paradoxical behaviors through case analyses or conducting situational simulations about dealing with innovation paradox in diverse teams can be beneficial in this regard.

Second, our results also highlight the impact of team perspective taking. Thus, we suggest that organizations should also encourage it. A very promising route might be for organizations to build cultures and climates that emphasize team perspective taking, for example, by stressing its value and importance in their interactions with team members, or even anchoring this perspective in the teams’ visions. Moreover, our findings further suggest that paradoxical leadership positively influences team perspective taking. Thus, it is valuable for leaders to be aware of their impacts on the team perspective taking. In particular, they are responsible for setting an example to guide team members on embracing differences by considering others’ perspectives, reframing their perceptions, and reinterpreting events. Meanwhile, leaders can implement training interventions and encourage team members to appreciate others’ viewpoints, and take the initiative to engage in team communication, collaboration, and other collective activities.

### Limitations and Future Directions

Despite our theoretical and practical implications, this study has some limitations. First, although it examines the boundary conditions of the association between expertise diversity and team innovative performance and tests the mediated moderating effects simultaneously, other mechanisms could also account for this managerial phenomenon. For example, team members adjust their behaviors not only according to team context, but also organizational practice. Factors associated with specific organizational practices, such as high-performance work systems, may also play a vital role in the team’s informational processes. Therefore, future studies could broaden our research findings by considering organizational-level determinants.

Second, although we measured variables from different sources (i.e., team members and leaders) at different times, it is still difficult to draw definitive causal conclusions from our findings because many of the relationships were likely reciprocally causal over time. For instance, team innovative performance might affect leader behaviors in managing the innovation process. To address causality, future research should use quasi-experimental or longitudinal designs. Additionally, we were unable to assess objective measures of team innovative performance. Accordingly, studies based on more objective team innovative performance measures would ensure more robust conclusions.

Third, we operationalized expertise diversity as an independent variable in terms of Blau’s index (Blau, 1977). However, previous studies show that diversity measured in terms of disparity or separation may also affect team innovative performance (Harrison and Klein, 2007; Chiocchio and Essiembre, 2009). Moreover, recent studies operationalize team composition in terms of fault lines that split teams into relatively homogenous subgroups and show that strong fault lines brought knowledge- and decision-related benefits (Nishii and Goncalo, 2008). In this case, future studies could capture team diversity as disparity or fault lines and investigate their impacts on team innovative performance.

## Ethics Statement

This study was carried out in accordance with the recommendations of ethics committee of Tsinghua University with written informed consent from all subjects. All subjects gave written informed consent in accordance with the Declaration of Helsinki. The protocol was approved by the ethics committee of Tsinghua University.

## Author Contributions

QL led the literature review, research design, data analysis, and paper drafting work for this paper. ZS made contributions in data analysis, paper revision during the review process. BY made contributions in data collection and paper drafting.

## Conflict of Interest Statement

The authors declare that the research was conducted in the absence of any commercial or financial relationships that could be construed as a potential conflict of interest.
